# LysoPC-acyl C16:0 is associated with brown adipose tissue activity in men

**DOI:** 10.1007/s11306-017-1185-z

**Published:** 2017-03-03

**Authors:** Mariëtte R. Boon, Leontine E. H. Bakker, Cornelia Prehn, Jerzy Adamski, Maarten J. Vosselman, Ingrid M. Jazet, Lenka M. Pereira Arias-Bouda, Wouter D. Marken van Lichtenbelt, Ko Willems van Dijk, Patrick C. N. Rensen, Dennis O. Mook-Kanamori

**Affiliations:** 1 Department of Medicine, Division of Endocrinology, Leiden University Medical Center, P.O. Box 9600, 2300 RC Leiden, The Netherlands; 2 Einthoven Laboratory for Experimental Vascular Medicine, Leiden University Medical Center, Leiden, The Netherlands; 3 Department of Human Biology, NUTRIM School for Nutrition, Toxicology and Metabolism, Maastricht University Medical Center, Maastricht, The Netherlands; 4 Institute of Experimental Genetics, Genome Analysis Center, Helmholtz Zentrum München, Neuherberg, Germany; 5 German Center for Diabetes Research, Neuherberg, Germany; 6 Lehrstul für Experimentelle Genetik, Technische Universität München, Freising-Weihenstephan, Germany; 7 Department of Radiology, Division of Nuclear Medicine, Leiden University Medical Center, Leiden, The Netherlands; 8 Department of Nuclear Medicine, Alrijne ziekenhuis, Leiderdorp, The Netherlands; 9 Department of Human Genetics, Leiden University Medical Center, Leiden, The Netherlands; 10 Department of Clinical Epidemiology, Leiden University Medical Center, Leiden, The Netherlands

**Keywords:** [^18^F]FDG PET-CT scan, Brown adipose tissue, LysoPC-acyl C16:0, Metabolomics

## Abstract

**Introduction:**

Brown adipose tissue (BAT) recently emerged as a potential therapeutic target in the treatment of obesity and associated disorders due to its fat-burning capacity. The current gold standard in assessing BAT activity is [^18^F]FDG PET-CT scan, which has severe limitations including radiation exposure, being expensive, and being labor-intensive. Therefore, indirect markers are needed of human BAT activity and volume.

**Objective:**

We aimed to identify metabolites in serum that are associated with BAT volume and activity in men.

**Methods:**

We assessed 163 metabolites in fasted serum of a cohort of twenty-two healthy lean men (age 24.1 (21.7–26.6) years, BMI 22.1 (20.5–23.4) kg/m^2^) who subsequently underwent a cold-induced [^18^F]FDG PET-CT scan to assess BAT volume and activity. In addition, we included three replication cohorts consisting of in total thirty-seven healthy lean men that were similar with respect to age and BMI compared to the discovery cohort.

**Results:**

After correction for multiple testing, fasting concentrations of lysophosphatidylcholine-acyl (LysoPC-acyl) C16:1, LysoPC-acyl C16:0 and phosphatidylcholine-diacyl C32:1 showed strong positive correlations with BAT volume (β= 116 (85–148) mL, R^2^ = 0.81, p = 4.6 × 10^−7^_;_ β = 79 (93–119) mL, R^2^ = 0.57, p = 5.9 × 10^−4^ and β= 91 (40–141) mL, R^2^ = 0.52, p = 1.0 × 10^−3^, respectively) as well as with BAT activity (β= 0.20 (0.11–0.29) g/mL, R^2^ = 0.59, p = 1.9 × 10^−4^; β = 0.15 (0.06–0.23) g/mL, R^2^ = 0.47, p = 2.0 × 10^−3^ and β= 0.13 (0.01–0.25) g/mL, R^2^ = 0.28, p = 0.04, respectively). When tested in three independent replication cohorts (total n = 37), the association remained significant between LysoPC-acyl C16:0 and BAT activity in a pooled analysis (β= 0.15 (0.07–0.23) g/mL, R^2^ = 0.08, p = 4.2 × 10^−4^).

**Conclusions:**

LysoPC-acyl C16:0 is associated with BAT activity in men. Since BAT is regarded as a promising tool in the battle against obesity and related disorders, the identification of such a noninvasive marker is highly relevant.

**Electronic supplementary material:**

The online version of this article (doi:10.1007/s11306-017-1185-z) contains supplementary material, which is available to authorized users.

## Introduction

In humans, at least two types of adipose tissue are present, white adipose tissue (WAT) and beige or brown adipose tissue (BAT). While the main function of WAT is the storage of fatty acids as triglycerides, BAT mainly oxidizes triglyceride-derived fatty acids to generate heat in response to various physiological stimuli including cold exposure (Cannon and Nedergaard 2004). Brown adipocytes contain a wealth of mitochondria containing uncoupling protein 1 (UCP-1) that uncouples respiration from adenosine 5′ triphosphate (ATP) synthesis by allowing leakage of protons over the mitochondrial inner membrane, leading to heat production (Cannon and Nedergaard 2004).

Interestingly, recent studies have shown that activation of BAT by means of cold or capsinoids recruits BAT in relation to an increase in whole-body energy expenditure and reduces fat mass in healthy individuals (van der Lans et al. 2013; Yoneshiro et al. 2012, 2013). Moreover, repetitive cold exposure improved glucose metabolism in patients with type 2 diabetes (Hanssen et al. 2015). Thus, albeit that the precise long term contribution of BAT to human energy expenditure remains to be determined, stimulation of BAT activity or ‘recruitment’ of BAT is currently considered a potential preventive and therapeutic target in the combat against obesity and related diseases, such as dyslipidemia (Hoeke et al. 2016) and type 2 diabetes mellitus (T2DM).

The current ‘gold standard’ for determination of BAT volume and BAT activity in humans is cold-induced [^18^F]fluorodeoxyglucose ([^18^F]FDG) positron emission tomography-computed tomography (PET-CT) (van der Lans et al. 2014). With this method, individuals are cooled down towards their shivering temperature, then stably cooled for 1 h at a temperature just above their shivering temperature to ensure maximum non-shivering thermogenesis, followed by infusion of [^18^F]FDG. After another hour of stable cooling, a PET-CT scan is performed (van Marken Lichtenbelt et al. 2009). Uptake of [^18^F]FDG by activated BAT regions on the PET-CT scan can be quantified, resulting in measures for BAT volume as well as for BAT activity.

The use of [^18^F]FDG PET-CT scans to assess BAT volume and activity is limited by their high costs and radiation burden. Therefore, measures of BAT volume and activity have yet to be performed in large population-based studies. Identification of novel, less invasive, labor-intensive or expensive, methods to assess BAT volume and activity is warranted. BAT is a metabolically highly active tissue, even at room temperature, and rapidly takes up glucose and triglyceride-derived fatty acids for combustion. As a consequence, metabolites that are produced or consumed due to BAT activity may change in plasma and thus, BAT volume and/or activity may be predicted by means of metabolites in serum. Therefore, the aim of the current study was to identify metabolites in a non-cooled state that are associated with BAT volume and/or activity in men.

## Methods

### Study population discovery cohort

Our discovery cohort consisted of twenty-four healthy male Caucasian (n = 12) and South Asian (n = 12) participants from a study that was performed previously in the Alrijne hospital (Leiderdorp, the Netherlands) between March and June 2013 (Bakker et al. 2014). This study was powered on the identification of a difference in BAT volume, resulting in inclusion of twelve subjects per group. Subjects were lean (BMI < 25 kg/m^2^) and healthy males aged 18–30 years that were enrolled via local advertisements. Subjects underwent a medical screening including their medical history, a physical examination, blood chemistry tests, and an oral glucose tolerance test (OGTT) to exclude individuals with T2DM according to the American Diabetes Association (ADA) 2010 criteria. Other exclusion criteria were rigorous exercise, smoking, and recent body weight change. Data from two individuals were removed from the analyses due to virtually absent BAT volume (0–1 mL) as assessed by [^18^F]FDG PET-CT, leaving twenty-two participants. The present study was approved by the Medical Ethical Committee of the Leiden University Medical Center and performed in accordance with the principles of the revised Declaration of Helsinki. All volunteers gave written informed consent before participation.

### Study population replication cohorts

To verify whether the associations were sufficiently robust, we included three replication cohorts consisting of in total forty-two healthy Caucasian men. These participants were derived from three studies performed at the Maastricht University Medical Centre (Maastricht, the Netherlands) between 2011 and 2014 (Vosselman et al. 2012, 2013, 2015). In study 1, (Vosselman et al. 2015), aimed at studying the effect of exercise on BAT volume and activity, twelve healthy lean endurance-trained and twelve lean sedentary male Caucasian men aged 18–35 years were included. Endurance-trained athletes were included in the trained group when they performed endurance exercise at least three times a week for the last 2 years, and had a maximal oxygen consumption (VO2_max_) of >55 mL/min/kg. The sedentary males were included in the untrained group if they did not perform more than 1 h of exercise per week for the last 2 years and had a VO2_max_ of <45 mL/min/kg. General exclusion criteria were use of medication, smoking, weight gain/loss of >3 kg in the last 6 months, hypertension and (family history of) diabetes. The trained men were included in replication cohort 1 and the untrained men in replication cohort 2.

Replication cohort 3 consisted of subjects derived from two studies (Vosselman et al. 2012, 2013) that had the same inclusion criteria and cooling protocol. These studies aimed at studying the effect of β-adrenergic stimulation on BAT (Vosselman et al. 2012) and the effect of a high-calorie meal on BAT (Vosselman et al. 2013). In these studies, in total 19 healthy male Caucasian subjects aged between 18 and 35 years were enrolled. All subjects were screened for medical history. Cardiovascular status was screened by means of an electrocardiogram and blood pressure measurement. All subjects had normal blood glucose levels.

In the replication cohorts, data from five participants were removed due to absence of [^18^F]FDG uptake in the BAT region, leaving thirty-seven participants in the replication set. For the pooled analyses, fifty-nine participants were included in the analyses.

All replication studies were approved by the medical ethical committee of the Maastricht University Medical Center and all subjects were treated according to the principles of the revised declaration of Helsinki.

### Study set-up discovery cohort

Individuals were studied in the morning after a 10-h overnight fast and after 24 h without exercise. Subsequently, they were exposed to a thermoneutral temperature (32 °C) for 60 min, after which a basal blood serum sample was taken in which metabolites were also measured (see below). To activate BAT, an individualized cooling protocol was applied. In short, subjects lay on a bed sandwiched between two water-perfused cooling mattresses (Blanketrol III, Cincinnati Sub-Zero Products, Cincinnati, OH, USA). Cooling started from 32 °C and temperature was gradually decreased until shivering occurred (after 50–60 min). Temperature was then raised by 3–4 °C and the cooling period of 2 h was started (defined as t_cold_ = 0 min). If shivering occurred, the temperature was raised in steps of 1 °C until shivering stopped. Shivering was detected visually or was reported by participants. After 1 h of cooling (t_cold_=60 min), 2 MBq/kg [^18^F]FDG was injected intravenously and subjects were instructed to lay still in order to prevent artifact by muscle activity. At t_cold_ = 110 min, a cold-induced blood sample was taken in which metabolites were measured as well. After 2 h of cooling (t_cold_=120 min) the PET-CT scan (Gemini TF PET-CT, Philips, The Netherlands) was performed to assess BAT volume and activity. Imaging was performed in three dimensional mode, with emission scans of 3 min per bed position in the upper part of the body (first seven bed positions) and scans of 30 s per bed position in the body area below, as described previously (Bakker et al. 2014). We quantified BAT activity and detectable volume in the region of interest by autocontouring the BAT areas with a set threshold (SUV) of 2.0 g/mL using Hermes software (Hermes Hybrid Viewer, Hermes Medical Solutions, Sweden).

### Study set-up replication cohorts

In all replication cohorts, subjects were studied in the morning after an overnight fast and after 24 h without exercise. All experiments started with 1-h baseline measurements during thermoneutral conditions (24–25 °C), after which a basal blood serum sample was taken in which metabolites were also measured (see below). Subsequently, subjects were exposed to 2 h of mild cold exposure in which an individualized protocol was used by means of air cooling using air-conditioning. In short, each subject was cooled down until shivering occurred (approximately after 20–60 min). After that, air temperature was slightly increased until shivering stopped. After 1 h of cold exposure the [^18^F]FDG tracer was injected intravenously (74 MBq in replication cohorts 1 and 2 and 50 MBq in replication cohort 3) and subjects were exposed to another hour of cold. Next, after 2 h of cooling, the PET-CT scan (Gemini TF PET-CT, Philips, the Netherlands) was performed to assess BAT volume and activity. Imaging was performed with emission scans of 6 min per bed position in the upper part of the body (six to seven bed positions) only. In replication cohort 1 and 2, BAT activity and detectable volume were quantified in the region of interest by autocontouring the BAT areas with a set threshold (SUV) of 1.5 g/mL (Vosselman et al. 2015). In replication cohort 3 a threshold of 1.5 g/mL was used as well, and additionally the regions drawn were localized in fat tissues only as determined by CT scan (HU −10 to −180) (Vosselman et al. 2012). For all analyses, PMOD software (PMOD Technologies) was used.

### Targeted metabolomics

Metabolomic measurements were performed on serum samples taken under thermoneutral and cold conditions at the Genome Analysis Center at the Helmholtz Zentrum, Munich, Germany, using the Biocrates Absolute*IDQ*™ p150 kit (BIOCRATES Life Science AG, Innsbruck, Austria) and ESI-FIA-MS/MS measurements (Menni et al. 2013). The assay allows simultaneous quantification of 163 small molecule metabolites within 10 µL serum. The assay includes free carnitine, 40 acylcarnitines, 14 amino acids (13 proteinogenic + ornithine), hexoses (of which approx. 90–95% is glucose), 92 glycerophospholipids including 15 lysophosphatidylcholines (LysoPCs) and 77 phosphatidylcholines (PCs), and 15 sphingolipids (Menni et al. 2013; Illig et al. 2010). Quantification of the metabolites was achieved by reference to appropriate internal standards. The assay has been previously described (Menni et al. 2013; Illig et al. 2010).

### Statistical analyses

First, all metabolites were Z-score transformed (i.e. cohort and ethnic-specific Z-scores) to make effect estimates between metabolites comparable. For the discovery cohort, multivariate linear regression analyses were used in both ethnicities separately to examine the associations between metabolites and BAT volume/activity, adjusting for age and body mass index. Pooled analyses for both ethnicities were performed by additionally adjusting the model for ethnicity. A p-value of 1.5 × 10^−4^ (= 0.05/(163 × 2) (163 metabolites tested against BAT volume and BAT activity)) was considered statistically significant.

For replication, we selected the one metabolite that reached the Bonferroni level of statistical significance and the two next ranked metabolites that were highly correlated to the top metabolite. Again, multivariate linear regression analyses were used to examine the associations between metabolites and BAT volume/activity, adjusting for age and body mass index. Pooled analysis for the replication was also achieved by additionally adjusting the model for the study set. For the replication, a p-value of 8.3 × 10^−3^ (= 0.05/(3 × 2) (3 metabolites tested against BAT volume and BAT activity)) was considered to be statistically significant. Finally, we performed pooled analyses for these three metabolites from the five sub-studies (n = 59 participants). To this end, multivariate linear regression analyses were used (adjusting for age, body mass index and study set) and the same p-value of 8.3 × 10^−3^ was considered statistically significant. To compare levels of metabolites at thermoneutral temperature and after cold exposure in the discovery cohort, paired T-tests were used. All statistical analyses were performed in Stata 12.1 (StataCorp, Texas, USA) and the SPSS 20 (SPSS Inc, Chicago, IL, USA).

## Results

### Participant characteristics

The characteristics of the discovery cohort are shown in Table 1. All participants were male and mean age was 24.1 years (range 21.7–26.6), while mean body mass index (BMI) was 22.1 kg/m^2^ (range 20.5–23.4). Mean BAT volume was 230 mL (range 158–299) and mean BAT activity expressed as mean standardized uptake value (SUV_mean_) 4.17 g/mL (range 4.01–4.36). As compared to the discovery cohort, the participants from the replication cohorts were similar with respect to gender, age and BMI. However, the participants from replication cohort 2 and 3 had higher detectable BAT volume (802 and 480 mL vs 230 mL, respectively, both p < 0.05) and lower BAT activity (2.52 and 2.35 g/mL vs 4.17 g/mL, respectively, both p < 0.05) as compared to the discovery cohort.

**Table 1:** Participant characteristics of the discovery and replication studies

	Discovery Bakker et al. 2014	Replication 1 Vosselman et al. 2015	Replication 2 Vosselman et al. 2015	Replication 3 Vosselman et al. 2012, 2013
Age (years)	24.1 (21.7–26.6)	25.7 (22.0–32.0)	22.8 (19.0–24.5)	22.8 (21.0–24.0)
N (% males)	22 (100%)	11 (100%)	10 (100%)	16 (100%)
Body mass index (kg/m^2^)	22.1 (20.5–23.4)	21.2 (19.5–22.5)	22.1 (21.3–23.4)	22.1 (20.7–23.3)
Ethnicity (n of participants)				
White Caucasian (%)	10 (46%)	11 (100%)	10 (100%)	16 (100%)
South Asian (%)	12 (54%)	0 (0%)	0 (0%)	0 (0%)
Brown adipose tissue volume (mL)	230 (158–299)	288 (164–480)	802 (551–1010)*	480 (167–766)*
Brown adipose tissue activity (SUV_mean_, g/mL)	4.17 (4.01–4.36)	2.12 (1.79–2.34)	2.52 (2.33–2.90)	2.35 (2.09–2.64)

Values represent number of participants (%) or median (interquartile range). *p < 0.05 vs Discovery study

### The metabolites LysoPC-acyl C16:1, LysoPC-acyl C16:0 and PC-diacyl C32:1 highly correlate with BAT volume and activity in the discovery cohort

**Fig. 1:**
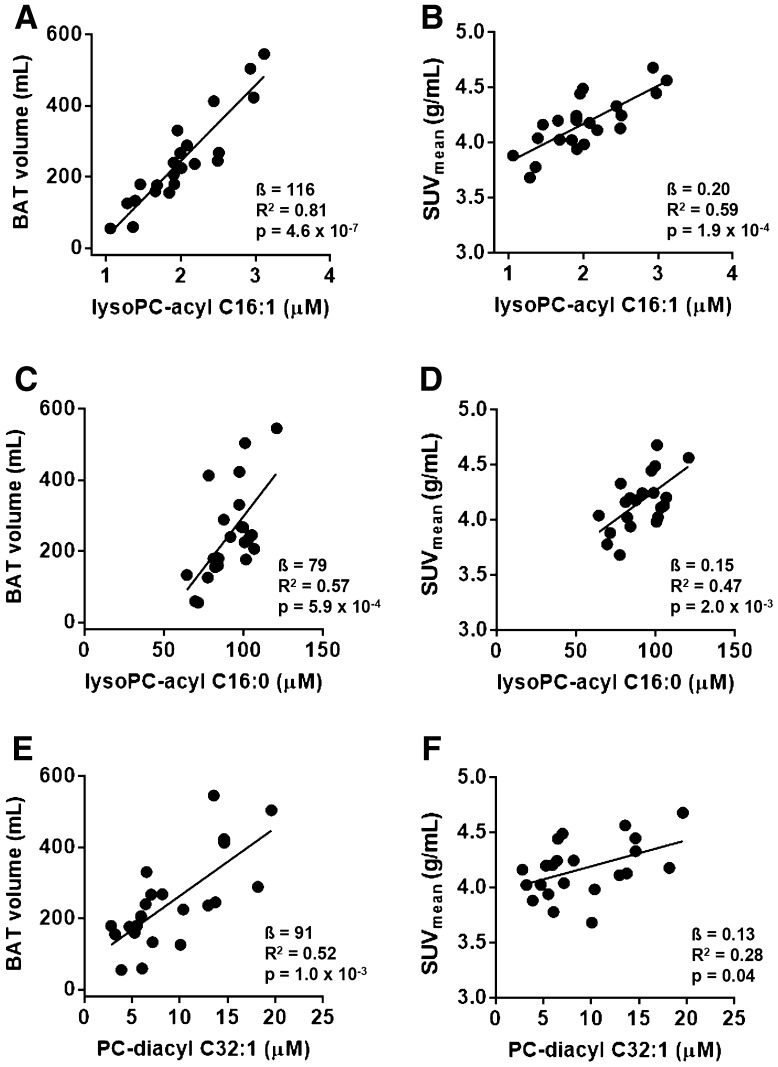
*Scatter plot* of the associations between lysophosphatidylcholine-acyl C16:1 (lysoPC-acyl C16:1) with brown adipose tissue (BAT) volume (**a**) and **b** BAT activity (SUV_mean_), between lysophosphatidylcholine-acyl C16:0 (lysoPC-acyl C16:0) with BAT volume (**c**) and BAT activity (**d**) and between phosphatidylcholine-diacyl C32:1 (PC-diacyl C32:1) with BAT volume (**e**) and BAT activity (**f**) in the discovery cohort (n = 22)

In search for metabolites in serum that are associated with BAT volume and activity, we measured 163 small molecule metabolites in fasting serum of the participants from our discovery cohort and correlated their concentrations to BAT volume and activity using multivariate linear regression (Suppl Table SI). After correction for multiple testing, fasting concentrations of the metabolite LysoPC-acyl C16:1 strongly positively associated with BAT volume (β= 116 (85–148) mL, R^2^ = 0.81, p = 4.6 × 10^− 7^) (Fig. 1a) as well as BAT activity (β= 0.20 (0.11–0.29) g/mL, R^2^ = 0.59, p = 1.9 × 10^− 4^) (Fig. 1b). The estimated effect on BAT volume was 116 mL per standard deviation increase (95% CI: 85, 148). These associations were found in both Caucasian individuals (p = 2.2 × 10^− 3^ for BAT volume and p = 3.0 × 10^− 3^ for BAT activity, respectively) and South Asian individuals (p = 2.4 × 10^− 3^ for BAT volume and p = 0.06 for BAT activity, respectively). Also, its related metabolites LysoPC-acyl C16:0 (Fig. 1c) and PC-diacyl C32:1 (Fig. 1e) correlated positively with BAT volume (β= 79 (93–119) mL, R^2^ = 0.57, p = 5.9 × 10^− 4^ and ß=91 (40–141) mL, R^2^ = 0.52, p = 1.0 × 10^− 3^, respectively). Furthermore, LysoPC-acyl C16:0 (Fig. 1d) and PC-diacyl C32:1 (Fig. 1f) correlated with BAT activity (β= 0.15 (0.06–0.23) g/mL, R^2^ = 0.47, p = 2.0 × 10^− 3^ and β= 0.13 (0.01–0.25) g/mL, R^2^ = 0.28, p = 0.04, respectively).

### Cold exposure tends to increase serum levels of LysoPC-acyl C16:1 and LysoPC-acyl C16:0 and increases LysoPC-diacyl C32:1 in the discovery cohort

**Fig. 2:**
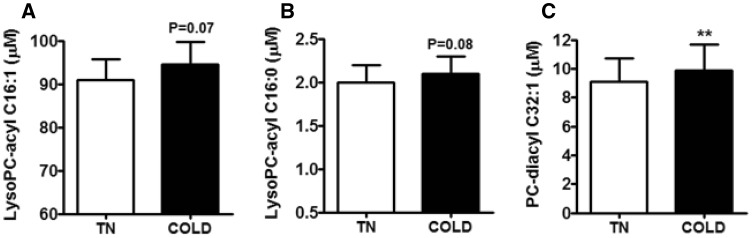
Effect of cold exposure (COLD) as compared to thermoneutrality (TN) on serum levels of lysophosphatidylcholine-acyl C16:1 (lysoPC-acyl C16:1) (**a**), lysophosphatidylcholine-acyl C16:0 (lysoPC-acyl C16:0) (**b**) and phosphatidylcholine-diacyl C32:1 (PC-diacyl C32:1) (**c**). Data are expressed as mean ± SEM and data were calculated by paired T-tests

To assess whether BAT activation directly influences levels of the three metabolites, we measured their levels in serum of our participants also after 2 h of stable cooling. Indeed, cooling tended to slightly increase levels of LysoPC-acyl C16:1 (+5%, p = 0.07, Fig. 2a) and LysoPC-acyl C16:0 (+4%, p = 0.08, Fig. 2b), albeit that the increase was small. Moreover, cold exposure slightly increased PC-diacyl C32:1 levels (+9%, p = 0.007, Fig. 2c).

### LysoPC-acyl C16:0 correlates with BAT activity in the replication cohorts

**Fig. 3:**
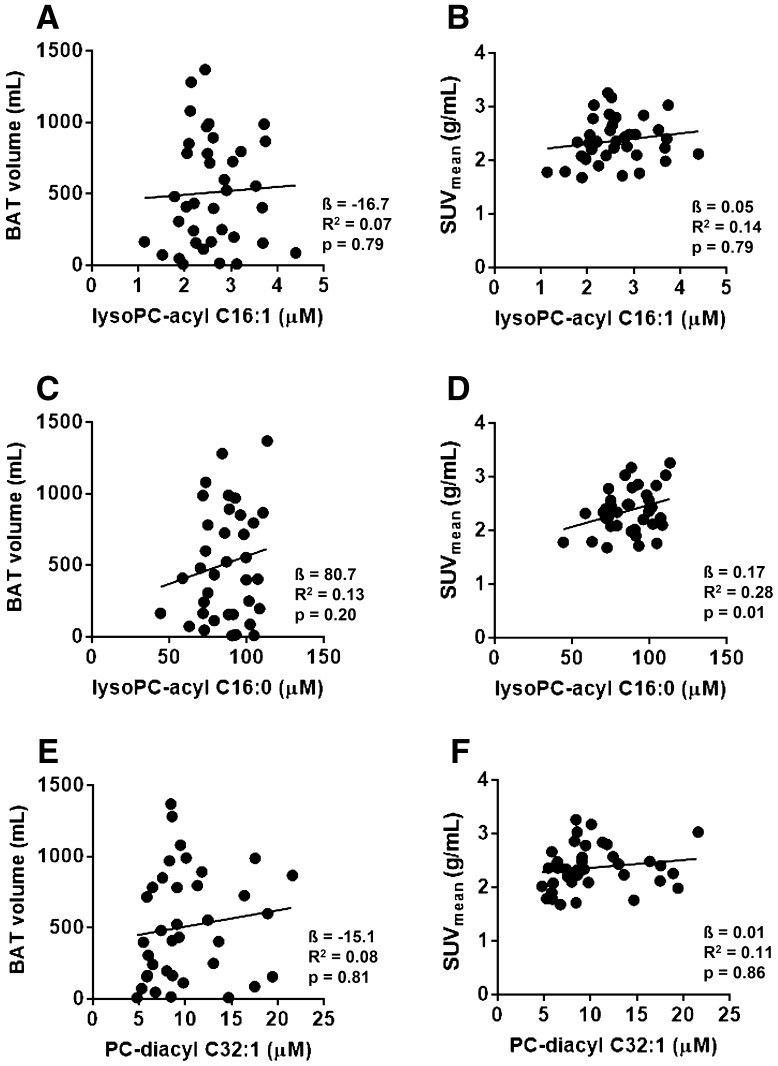
*Scatter plot* of the associations between lysophosphatidylcholine-acyl C16:1 (lysoPC-acyl C16:1) with brown adipose tissue (BAT) volume (**a**) and BAT activity (SUV_mean_) (**b**), between lysophosphatidylcholine-acyl C16:0 (lysoPC-cyl C16:0) with BAT volume (**c**) and BAT activity (**d**) and between phosphatidylcholine-diacyl C32:1 (PC-diacyl C32:1) with BAT volume (**e**) and BAT activity (**f**) in the pooled replication cohorts (n = 37)

We next studied whether these three metabolites were sufficiently robust by performing a replication study in cohorts from a different academic center. Albeit that we could not reproduce the correlation between LysoPC-acyl C16:1 and LysoPC-diacyl C32:1 and BAT volume and activity (Fig. 3), the effect estimates for LysoPC-acyl C16:0 were all in the same direction (positive) as the discovery set (Table 2). In the replication, the pooled analysis for the correlation between LysoPC-acyl C16:0 and BAT activity was nominally significant (p < 0.05), but fell slightly short of reaching statistical significance after Bonferroni correction (p = 0.01) (Table 2; Fig. 3d). However, when we assessed with the pooled association of BAT volume and activity in all 59 participants (as shown in Table 2), LysoPC-acyl C16:0 was significantly correlated with BAT activity (β= 0.15, R^2^ = 0.08, p = 4.2 × 10^−4^) and the estimated effect on BAT activity was 0.15 per standard deviation increase (95% CI: 0.07, 0.23).

**Table 2:** Associations between LysoPC-acyl C16:1, LysoPC-acyl C16:0, and PC-diacyl 32:1 and brown adipose tissue (BAT) volume and BAT activity

	BAT Volume			BAT Activity		
	LysoPC-acyl C16:1	LysoPC-acyl C16:0	PC-diacyl C32:1	LysoPC-acyl C16:1	LysoPC-acyl C16:0	PC-diacyl C32:1
Discovery (white Caucasians)	102.2 (53.2, 151.3) p = 0.002	65.6 (−22.0, 153.3) p = 0.12	72.9 (−5.3, 151.2) p = 0.06	0.14 (0.07, 0.21) p = 0.003	0.09 (−0.03, 0.21) p = 0.11	0.09 (−0.02, 0.20) p = 0.08
Discovery (South Asians)	99.3 (46.8, 151.9) p = 0.002	60.4 (19.5, 101.3) p = 0.009	77.2 (−6.2, 160.6) p = 0.07	0.18 (−0.01, 0.37) p = 0.06	0.12 (−0.01, 0.25) p = 0.07	0.05 (−0.21, 0.30) p = 0.68
Pooled analyses discovery	116.3 (85.1, 147.5) p = 4.6 × 10^− 7^	78.9 (39.3, 118.5) p = 5.9 × 10^− 4^	90.7 (40.1, 141.4) p = 0.001	0.20 (0.11, 0.29) p = 1.9 × 10^− 4^	0.15 (0.06, 0.23) p = 0.002	0.13 (0.01, 0.25) p = 0.04
Replication 1	−11.5 (−224.2, 201.2) p = 0.90	106.7 (−163.6, 376.9) p = 0.38	−1.6 (−264.0, 260.1) p = 0.99	0.09 (−0.12, 0.29) p = 0.34	0.23 (0.02, 0.44) p = 0.04	0.09 (−0.17, 0.34) p = 0.45
Replication 2	−6.3 (−439.7, 313.1) p = 0.70	110.1 (−173.4, 393.5) p = 0.38	−52.0 (−318,4, 214.4) p = 0.65	−0.10 (−0.48, 0.28) p = 0.56	0.24 (0.04, 0.44) p = 0.03	−0.09 (−0.36, 0.17) 0.43
Replication 3	−21.4 (−348.5, 305.7) p = 0.89	26.4 (−263.9, 316.7) p = 0.85	−12.9 (−387.7, 361.9) p = 0.94	0.07 (−0.31, 0.46) p = 0.69	0.08 (−0.26, 0.42) p = 0.61	0.06 (−0.38, 0.50) p = 0.78
Pooled analyses replication	−16.7 (−140.1, 107.3) p = 0.79	80.7 (−45.6, 207.0) p = 0.20	−15.1 (−142.7, 112.4) p = 0.81	0.05 (−0.09, 0.18) p = 0.49	0.17 (0.04, 0.30) p = 0.01	0.01 (−0.13, 0.15) p = 0.86
Pooled analyses all studies	21.1 (−60.0, 102.1) p = 0.60	72.0 (−3.2, 147.1) p = 0.06	22.2 (−59.8, 104.1) p = 0.59	0.09 (0.00, 0.18) p = 0.05	0.15 (0.07, 0.23) p = 4.2 × 10^− 4^	0.05 (−0.05, 0.14) p = 0.35

Values represent effect estimates (mL for volume, g/mLfor activity expressed as SUV_mean_) per standard deviation change in metabolite concentration. Model is adjusted for age and body mass index. In the pooled analyses, model is additionally adjusted for sub-study

## Discussion

In the search for metabolites in serum associated with BAT, we found that BAT volume and activity highly correlated with LysoPC-acyl C16:1 (p = 4.6 × 10^−7^ and p = 1.0 × 10^−6^), LysoPC-acyl C16:0 (p = 5.9 × 10^−4^ and p = 8.2 × 10^−4^) and PC-diacyl C32:1 (p = 1.5 × 10^−3^ and p = 1.3 × 10^−3^) in a cohort of young lean men, irrespective of ethnicity. Furthermore, BAT activation by means of cold exposure increased LysoPC-diacyl C32:1 and tended to increase serum levels of LysoPC-acyl C16:1 and LysoPC-acyl C16:0. Interestingly, the positive correlation between LysoPC-acyl C16:0 and BAT activity remained after pooling of four study cohorts (p = 4.2 × 10^−4^), suggesting that LysoPC-acyl C16:0 may potentially be a marker that predicts BAT activity in adult men.

To the best of our knowledge, this is the first study investigating the correlation between metabolites in a non-cooled state and BAT volume and activity in human adults. Since BAT is regarded a promising tool in the battle against obesity and related disorders and noninvasive tools to estimate its volume and activity are not available, the identification of such a serum metabolite is highly relevant. This can add in understanding the physiological role of human BAT and, furthermore, can be used in large population-based studies to study the effect of pharmacological activation of human BAT without the need for invasive measurements.

It is interesting to speculate on the mechanism by which LysoPC-acyl C16:0 levels may be linked with BAT. BAT is a metabolically active organ that efficiently internalizes fatty acids from lipoproteins (Bartelt et al. 2011), mostly derived from lipoprotein lipase (LPL)-mediated lipolysis of triglycerides (Khedoe et al. 2015). LPL-mediated processing of lipoproteins results in the generation of phospholipid-rich surface remnants that will intercalate into the high density lipoprotein (HDL) pool and accept cholesterol from peripheral tissues (Magill et al. 1982). Indeed, we recently showed that BAT activation strongly increases reverse cholesterol transport in mice (Bartelt et al. 2017), and that cold-induced BAT activation enhances the concentration of specifically small HDL particles and cholesterol levels within these small HDL particles in men (G Hoeke and K Nahon, *submitted*). The efflux of cholesterol is probably coupled to rapid lecithin:cholesterol acyltransferase (LCAT)-mediated esterification of cholesterol with a fatty acid that is liberated from PC resulting in the generation of LysoPC (Eisenberg 1983; Glomset 1968; Magill et al. 1982). Of note, we also recently showed that 2 h of cold exposure increases LysoPC-acyl C16:0 in the HDL pool of lean men, but not obese men (Bartelt et al. 2017). Together with the finding that cold exposure tended to increase serum levels of LysoPC-acyl C16:1 and LysoPC-acyl C16:0 this suggests that LysoPCs are generated as a consequence of BAT activity, albeit that we cannot exclude a contribution from other organs. For instance, white adipose tissue mobilization of fatty acids may, after processing, indirectly affect serum levels of LysoPC-acyl C16:1 and LysoPC-acyl C16:0. The fact that the increases in LysoPC-acyl C16:1 and LysoPC-acyl C16:0 did not reach statistical significance may be related to the relative short duration of cold exposure.

After pooling of four study cohorts only the correlation between LysoPC-acyl C16:0 and BAT activity remained significant. This may, at least in part, be due to the fact that the studies of the discovery cohort and replication cohorts were conducted in different medical centers. While in the discovery cohort water cooling was used to activate BAT, in the replication cohorts air cooling was used and this may have led to a submaximal activation of BAT. Although the amount of injected [^18^F]FDG was lower in the replication cohorts, it is unlikely that this accounts for the lower measured SUV_mean_ since this was compensated by an increase in scanning time per bed position in the replication cohorts compared to the discovery cohort (6 vs 3 min). Furthermore, the SUV threshold set for definition of BAT activity was lower (1.5 vs 2.0 g/mL) in the replication cohort, thus generally resulting in higher reported BAT volume. Indeed, reported BAT volume was markedly higher in replication cohorts 2 and 3 as compared to the discovery cohort. In replication cohort 3, for determination of BAT volume CT HU between −10 and −180 were used. This increases the selectivity for fat tissue but may result in underestimation of BAT volume (Van der Lans et al. 2014). However, as compared to the discovery cohort, BAT volume was actually increased in replication cohort 3, which is likely due to the lower BAT detection limit (e.g. 1.5 g/mL). Also, software used to analyze BAT volume and activity differed between discovery cohort and replication cohorts. This may have affected the quantitative measures of BAT activity (especially SUV_mean_) and volume. Furthermore, one of the replication cohorts included athletes. This may have increased heterogeneity in the replication cohort due to the fact that they had a slightly different body composition (lower fat mass) and lower BAT activity compared to the sedentary cohort (Vosselman et al. 2015). All of the above-mentioned methodological issues may have made our replication less robust. Still, despite these methodological issues, the pooled analyses showed that the correlation between LysoPC-acyl C16:0 and BAT activity remained significant. However, we do suggest for future studies and comparative reasons to keep thresholds and other settings equal between studies (Chen et al. 2016). Another potential limitation of our study is that we only included lean male participants in our study and, therefore, results may not apply to women nor obese subjects. Future studies should assess whether LysoPC-acyl C16:0 could also serve as a potential marker of BAT activity in these groups.

For our analyses, we excluded two participants with virtually absent BAT as assessed by PET-CT scan using the glucose tracer [^18^F]FDG. Of note, one of these participants had a very high [^18^F]FDG uptake in intercostal muscles (Bakker et al. 2014). Also, low [^18^F]FDG uptake does not exclude the presence of BAT and other biological mechanisms such as insulin resistance may underlie the low glucose uptake in these subjects. In fact, a recent study from Blondin et al. (Blondin et al. 2015) showed that glucose uptake by BAT does not correlate with uptake of [^18^F]FTHA (a measure of nonesterified fatty acid uptake) nor with [^11^C]acetate (a measure of the oxidative capacity of the tissue). Still, re-analysis of the correlation between BAT volume and LysoPC-acyl C16:1 in discovery population showed that the correlation was still highly significant (p = 1.2 × 10^−5^) when the BAT negative subject is included. Furthermore, in our study, we used a stringent Bonferroni correction, which assumes that all metabolites are independent from each other. However, many of the metabolites are in fact highly correlated to each other and, therefore, a Bonferroni correction is overly conservative. Finally, we found no evidence for heterogeneity (p = 0.42) for the association between LysoPC-acyl C16:0 and BAT activity, further underlining the robustness of our finding.

In conclusion, in the current study we show a robust positive correlation between serum LysoPC-acyl C16:0 levels and BAT activity. LysoPC-acyl C16:0 could potentially serve as a marker to predict BAT activity in a noninvasive way. Further larger studies, also encompassing a wider range of metabolites, are required to find additional predictors for BAT volume and activity.

## Electronic supplementary material

Below is the link to the electronic supplementary material.

Supplementary material 1 (XLSX 93 KB)

## Abbreviations

ATP: Adenosine 5’ triphosphate
BAT: Brown adipose tissue
BMI: Body mass index
FDG: Fluorodeoxyglucose
HDL: High density lipoprotein
LCAT: Lecithin:cholesterol acyltransferase
LPL: Lipoprotein lipase
LysoPC: Lysophosphatidylcholine
OGTT: Oral glucose tolerance test
PC: Phosphatidylcholine
PET-CT: Positron emission tomography-computed tomography
T2DM: Type 2 diabetes mellitus
UCP-1: Uncoupling protein-1
WAT: White adipose tissue

## References

[CR1] Bakker LE, Boon MR, van der Linden RA, Arias-Bouda LP, van Klinken JB, Smit F (2014). Brown adipose tissue volume in healthy lean south Asian adults compared with white Caucasians: A prospective, case-controlled observational study. The Lancet Diabetes & Endocrinology.

[CR2] Bartelt A, Bruns OT, Reimer R, Hohenberg H, Ittrich H, Peldschus K (2011). Brown adipose tissue activity controls triglyceride clearance. Nature Medicine.

[CR3] Bartelt, A., John, C., Schaltenberg, N., Berbée, J.F.P., Worthmann, A., Cherradi, M.L., et al. (2017). Thermogenic adipocytes promote HDL turnover and reverse cholesterol transport. *Nature Communications*. (in press) 10.1038/ncomms15010 PMC5399294 28422089

[CR4] Blondin DP, Labbe SM, Noll C, Kunach M, Phoenix S, Guérin B (2015). Selective impairment of glucose but not fatty acid or oxidative metabolism in brown adipose tissue of subjects with type 2 diabetes. Diabetes.

[CR5] Cannon B, Nedergaard J (2004). Brown adipose tissue: Function and physiological significance. Physiological Reviews.

[CR6] Chen KY, Cypess AM, Laughlin MR, Haft CR, Hu HH, Bredella MA (2016). Brown adipose reporting criteria in imaging studies (BARCIST 1.0): Recommendations for standardized FDG-PET/CT experiments in humans. Cell Metabolism.

[CR7] Eisenberg S (1983). Lipoproteins and lipoprotein metabolism. A dynamic evaluation of the plasma fat transport system. Wiener Klinische Wochenschrift.

[CR8] Glomset JA (1968). The plasma lecithins: Cholesterol acyltransferase reaction. The Journal of Lipid Research.

[CR9] Hanssen MJ, Hoeks J, Brans B, van der Lans AA, Schaart G, van den Driessche JJ (2015). Short-term cold acclimation improves insulin sensitivity in patients with type 2 diabetes mellitus. Nature Medicine.

[CR10] Hoeke G, Kooijman S, Boon MR, Rensen PC, Berbée JF (2016). Role of brown fat in lipoprotein metabolism and atherosclerosis. Circulation Research.

[CR11] Illig T, Gieger C, Zhai G, Römisch-Margl W, Wang-Sattler R, Prehn C (2010). A genome-wide perspective of genetic variation in human metabolism. Nature Genetics.

[CR12] Khedoe PP, Hoeke G, Kooijman S, Dijk W, Buijs JT, Kersten S (2015). Brown adipose tissue takes up plasma triglycerides mostly after lipolysis. The Journal of Lipid Research.

[CR13] Magill P, Rao SN, Miller NE, Nicoll A, Brunzell J, Hilaire St, J (1982). Relationships between the metabolism of high-density and very-low-density lipoproteins in man: Studies of apolipoprotein kinetics and adipose tissue lipoprotein lipase activity. European Journal of Clinical Investigation.

[CR14] Menni C, Zhai G, Macgregor A, Prehn C, Römisch-Margl W, Suhre K (2013). Targeted metabolomics profiles are strongly correlated with nutritional patterns in women. Metabolomics.

[CR15] Van der Lans AA, Hoeks J, Brans B, Vijgen GH, Visser MG, Vosselman MJ (2013). Cold acclimation recruits human brown fat and increases nonshivering thermogenesis. Journal of Clinical Investigation.

[CR16] Van der Lans AA, Wierts R, Vosselman MJ, Schrauwen P, Brans B, van Marken Lichtenbelt WD (2014). Cold-activated brown adipose tissue in human adults: Methodological issues. American Journal of Physiology Regulatory, Integrative and Comparative Physiology.

[CR17] Van Marken Lichtenbelt WD, Vanhommerig JW, Smulders NM, Drossaerts JM, Kemerink GJ, Bouvy ND (2009). Cold-activated brown adipose tissue in healthy men. The New England Journal of Medicine.

[CR18] Vosselman MJ, Brans B, van der Lans AA, Wierts R, van Baak MA, Mottaghy FM (2013). Brown adipose tissue activity after a high-calorie meal in humans. American Journal of Clinical Nutrition.

[CR19] Vosselman MJ, Hoeks J, Brans B, Pallubinsky H, Nascimento EB, van der Lans AA (2015). Low brown adipose tissue activity in endurance-trained compared with lean sedentary men. International Journal of Obesity (London).

[CR20] Vosselman MJ, van der Lans AA, Brans B, Wierts R, van Baak MA, Schrauwen P (2012). Systemic beta-adrenergic stimulation of thermogenesis is not accompanied by brown adipose tissue activity in humans. Diabetes.

[CR21] Yoneshiro T, Aita S, Kawai Y, Iwanaga T, Saito M (2012). Nonpungent capsaicin analogs (capsinoids) increase energy expenditure through the activation of brown adipose tissue in humans. American Journal of Clinical Nutrition.

[CR22] Yoneshiro T, Aita S, Matsushita M, Kayahara T, Kameya T, Kawai Y (2013). Recruited brown adipose tissue as an antiobesity agent in humans. Journal of Clinical Investigation.

